# Correlation between thyroid hormone sensitivity and the risk of polycystic ovary syndrome

**DOI:** 10.1186/s12902-024-01607-3

**Published:** 2024-05-30

**Authors:** Qian Wang, Ru Zhao, Chen Han, Zeyu Huang, Yan Bi, Xiaowen Zhang, Shanmei Shen

**Affiliations:** 1 Department of Endocrinology, Endocrine and Metabolic Disease Medical Center, Nanjing Drum Tower Hospital, Affiliated Hospital of Medical School, Nanjing University, Nanjing, China; 2 Branch of National Clinical Research Centre for Metabolic Diseases, Nanjing, China; 3 Department of Endocrinology, Endocrine and Metabolic Disease Medical Center, Nanjing Drum Tower Hospital Clinical College of Nanjing Medical University, Nanjing, China; 4 Department of Endocrinology and Metabolism, Drum Tower Clinical Medical College, Southeast University, Nanjing, China

**Keywords:** Sensitivity to thyroid hormones, Thyroid, Polycystic ovary syndrome, PCOS

## Abstract

**Objective:**

There has been some confusion in earlier research on the connection between thyroid function and polycystic ovary syndrome (PCOS). This research is aimed to probe into the correlation between thyroid condition and the risk of PCOS from a new standpoint of thyroid hormone sensitivity.

**Methods:**

This research comprised 415 females with PCOS from Drum Tower Hospital Affiliated with the Medical School of Nanjing University, and 137 non-PCOS individuals were selected as the normal control. Based on free thyroxine (FT4), free triiodothyronine (FT3), and thyroid-stimulating hormone (TSH), we calculated the thyroid hormone sensitivity indices, which consist of Thyroid Feedback Quantile-based Index (TFQI), Thyroid-stimulating Hormone Index (TSHI), Thyrotroph Thyroxine Resistance Index (TT4RI) and Free Triiodothyronine /Free thyroxine (FT3/FT4). The binary logistic regression model was adopted to investigate the correlation between thyroid hormone sensitivity indices with the risk of PCOS. Pearson or Spearman correlation analysis was employed to explore the association among thyroid-related measures with metabolic parameters in PCOS.

**Results:**

Results of this research showed that females with PCOS had rising TFQI, TSHI, TT4RI, and FT3/FT4 levels compared with the control group. After adjustment for the impact of various covariates, there was no significant correlation between FT3/FT4 and the risk of PCOS; However, the odds ratio of the third and fourth vs. the first quartile of TFQI were 3.57(95% confidence interval [CI]:1.08,11.87) and 4.90(95% CI:1.38,17.38) respectively; The odds ratio of the fourth vs. the first quartile of TSHI was 5.35(95% CI:1.48,19.37); The odds ratio of the second vs. the first quartile of TT4RI was 0.27(95%CI 0.09,0.82). In addition, no significant correlation was observed between thyroid-related measures and metabolic measures in females with PCOS.

**Conclusions:**

A reduction in the sensitivity of central thyroid hormone is closely correlated with a higher risk of PCOS. Further research is necessary to corroborate our findings and the supporting mechanisms.

## Introduction

As one of the most prevalent endocrine illnesses, polycystic ovary syndrome (PCOS) influences almost 5–10% of females of child-bearing age [1–3]. Menstrual disorders, anovulation infertility, androgen excess, and polycystic changes in ovaries are all symptoms of PCOS. In addition, it is also in connection with an added accident of obesity, insulin resistance (IR), Type 2 diabetes (T2D), metabolic syndrome (MS), cardiovascular disease, and cancer of the endometrium [1, 4–6].

Thyroid hormones are closely related to PCOS [7]. Multiple studies demonstrated that the prevalence of thyroid diseases was substantially greater in females with PCOS in comparison to age-matched controls [8–10]. For the past few years, a rising quantity of scholars has taken notice of the correlation between metabolic disorders and thyroid hormone sensitivity indexes which have been proven to be credible predictors of IR, T2D, hyperuricemia, cardiometabolic risk, and disturbances of lipid metabolism [11–14]. As a metabolic disease, however, PCOS has not been investigated concerning sensitivity to thyroid hormones. Therefore, we adopt four thyroid hormone sensitivity indices, including FT3/FT4, thyrotropin T4 resistance index (TT4RI), TSH index (TSHI), and thyroid feedback Quantile index (TFQI) [13–16], to look into the connection between thyroid conditions and PCOS.

## Patients and methods

### Study population

**Fig. 1:**
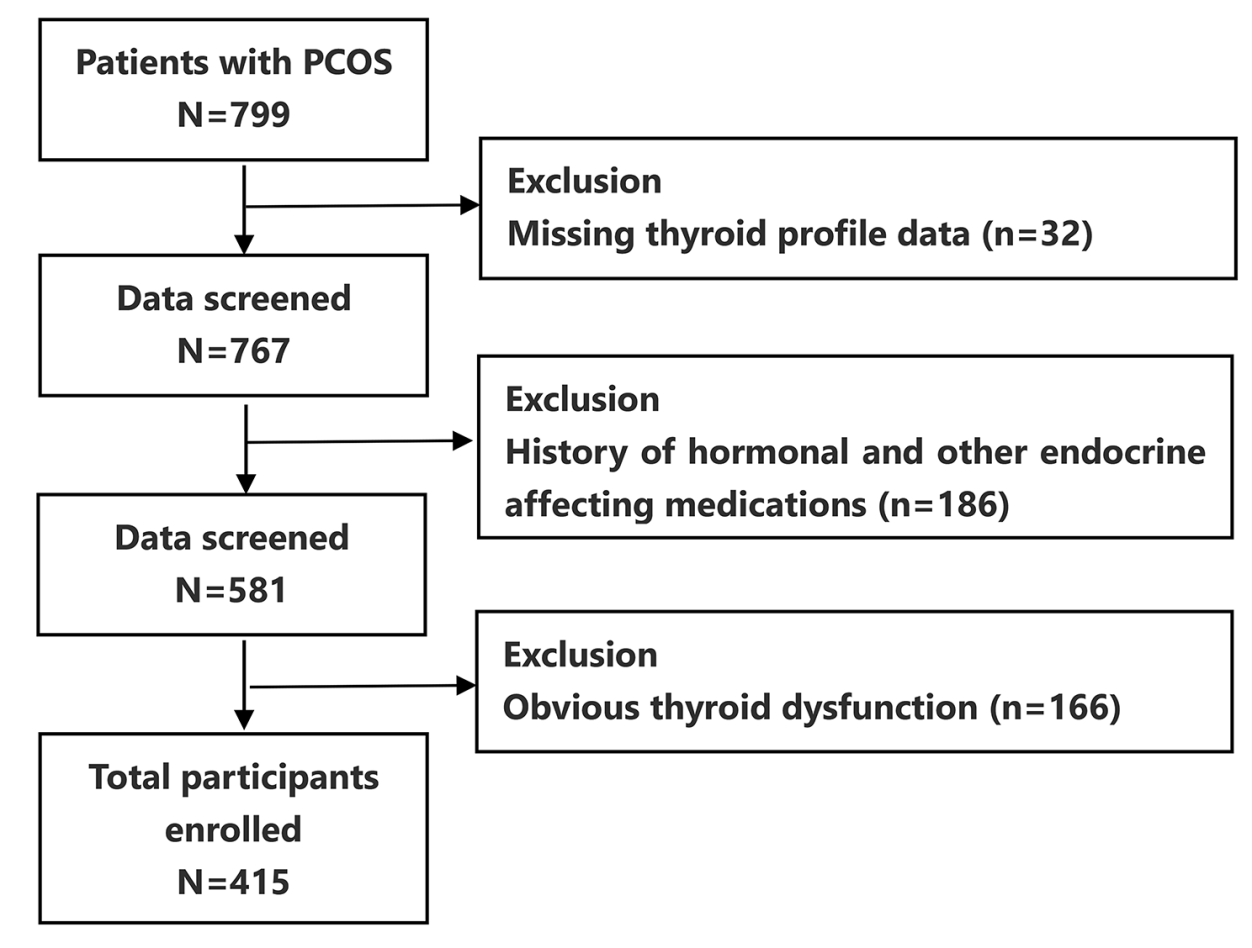
Flowchart of the inclusion and exclusion of participants

The study group consists of 415 females with PCOS who visited the PCOS Special Clinic of our hospital from May 2016 to February 2019 (Fig. 1). Inclusion criteria: (1) Definite diagnosis of PCOS, all patients were diagnosed by an experienced endocrinologist or gynecologist with expertise in the field and underwent our re-evaluation before enrollment; (2) Age 18–40 years old; (3) Has not been treated for PCOS. Exclusion criteria: (1) Use of hormones or added medications in the last three months that could exert an impact on endocrine metabolism; (2) The existence of significant thyroid dysfunction; (3) Pregnancy or lactation; (4) Severe cardiopulmonary dysfunction and liver and kidney insufficiency. Another 137 cases of concurrent health check-up females were chosen as the control group. Inclusion criteria: (1) Females aged 18–40 years; (2) Clinical information and examination indicators are complete. Exclusion criteria: Individuals with PCOS, obesity, hypertension, hyperlipidemia, and other metabolic diseases are excluded.

### Diagnostic criteria

The 2003 Rotterdam criteria revisions were used to diagnose PCOS [17]: (1). Oligo-ovulation and/or anovulation; (2). Hyperandrogenemia and/or clinical hyperandrogenic manifestations; (3). Polycystic changes in the ovaries; and exclusion of other diseases that may cause androgen excess such as Cushing’s syndrome.

### Data collection

General clinical information such as age, height, weight, and blood pressure levels was recorded. And calculate body mass index (BMI). Approximately 5 ml of venous blood was drawn from each subject in the morning, on days 2–5 of the menstrual cycle, following a 12-hour overnight fast. Calculate thyroid hormone sensitivity indices: FT3/FT4 ratio = FT3/FT4, which indicates that peripheral thyroid hormone sensitivity is elevated when FT3/FT4 is higher. TT4RI = FT4∗TSH, TSHI = lnTSH + 0.1345∗FT4, the central thyroid hormone sensitivity is inversely linked to the TT4RI and TSHI values. TFQI = cdfFT4-(1-cdfTSH), the coefficient of TFQI is achieved by applying the medical examination population. TFQI is superior because it does not generate extremes even under the circumstance of thyroid dysfunction when compared to TT4RI and TSHI. TFQI takes the amount from − 1 to 1. A positive TFQI implies regular insensitivity, while a negative TFQI shows that the HPT axis is more responsive to variation in FT4, and 0 indicates normal sensitivity.

### Laboratory tests

All laboratory parameters were measured at our hospital’s clinical laboratory, adhering to the ISO15189 international quality standard. The enzymatic auto-analyzer (Kyowa Medex Co., Ltd., Tokyo, Japan) was used to measure concentrations of Fasting Plasma Glucose (FPG), Triglyceride (TG), Total Cholesterol (TC), High-Density Lipoprotein Cholesterol (HDL-C), and Low-Density Lipoprotein Cholesterol (LDL-C), following the manufacturer’s instructions. Electrochemiluminescence (Roche Diagnostics, Basel, Switzerland) was used to measure serum levels of TSH, FT3, and FT4, according to standardized methods and rigorous quality control protocols. The reference ranges for each parameter are as follows. FPG, 3.9-6.1mmol/L; TG, ≤ 1.7mmol/L; TC, 2.9-5.72mmol/L; HDL-C, 0.94-2mmol/L; LDL-C, 1.89–3.1 mmol/L; TSH, 0.27–4.2 mIU/L; FT3, 3.1–6.8 pmol/L; FT4, 12–22 pmol/L.

### Statistical analyze

The data were examined by adopting the program SPSS 27.0. Normally distributed variables were expressed as means ± SD, otherwise, they are expressed as quartiles. T-test and Mann-Whitney test were adopted to compare the differences between groups, separately. The binary logistic regression analysis was used to examine the overall PCOS risk at the thyroid hormone sensitivity index quartile. For finding the correlation, Pearson’s correlation was applied to continuous variables with normal distribution, while Spearman’s correlation was applied to skewed distributed ones. Statistics were deemed significant when *P* < 0.05.

## Results

This research contained 552 participants, and their average age was 28 (SD 3.51 years); 415 were patients with PCOS, and 137 were from the healthy check-up population, with clinical characteristics shown in Table 1. Compared to the healthy check-up population, patients with PCOS had higher weight, BMI, blood pressure, FPG, TG, TC, LDL levels, lower HDL, and higher TSH, FT3, FT4, FT3/FT4, TT4RI, TSHI, TFQI levels (*P*<0.05).

**Table 1:** Baseline characteristics

	Overall	Healthy population	PCOS	*P* value
N	552	137	415	
Age	28.00 ± 3.51	28.13 ± 3.47	27.90 ± 3.42	0.604
Height (cm)	160.65 ± 5.07	160.50 ± 4.23	160.78 ± 5.37	0.646
Weight (kg)	74.10(61.60,83.70)	53.60(49.80,58.05)	78.70(72.03,86.28)	<0.001
BMI	29.30(24.22,32.00)	20.94(19.34,22.72)	30.35(28.57,32.80)	<0.001
SBP (mmHg)	118.96 ± 11.42	112.46 ± 10.09	121.28 ± 11.08	<0.001
DBP (mmHg)	76.74 ± 9.39	70.11 ± 8.25	79.07 ± 8.65	<0.001
FPG (mmol/L)	5.07(4.73,5.47)	4.69(4.47,4.92)	5.21(4.86,5.61)	<0.001
TG (mmol/L)	1.32(0.80,2.00)	0.69(0.57,0.92)	1.57(1.09,2.27)	<0.001
TC (mmol/L)	4.52(4.06,5.13)	4.34(3.86,4.86)	4.60(4.11,5.23)	<0.001
HDL-C (mmol/L)	1.12(0.93,1.40)	1.49(1.21,1.66)	1.06(0.89,1.24)	<0.001
LDL-C (mmol/L)	2.56(2.03,3.10)	2.07(1.78,2.47)	2.71(2.23,3.25)	<0.001
TSH (pmol/L)	2.54(1.81,3.30)	2.38(1.72,2.83)	2.62(1.84,3.48)	0.001
FT3 (pmol/L)	5.20 ± 0.64	4.76 ± 0.59	5.35 ± 0.59	<0.001
FT4 (pmol/L)	17.21 ± 1.97	16.75 ± 1.78	17.33 ± 1.99	0.002
FT3/FT4	0.30(0.27,0.33)	0.28(0.26,0.31)	0.31(0.28,0.34)	<0.001
TT4RI	42.35(30.62,56.38)	38.38(28.45,49.44)	44.65(31.70,60.33)	<0.001
TSHI	3.21 ± 0.40	3.04 ± 0.43	3.27 ± 0.50	<0.001
PTFQIFT4	0.07 ± 0.25	-0.02 ± 0.23	0.10 ± 0.24	<0.001

Continuous variables are presented as mean ± standard deviation (SD) or median (interquartile range)

Abbreviations: BMI: body mass index; SBP: systolic blood pressure; DBP: diastolic blood pressure; FPG: fasting plasma glucose level; TG: triglycerides; TC: total cholesterol; HDL-C: high-density lipoprotein cholesterol; LDL-C: low-density lipoprotein cholesterol; TSH: thyroid stimulating hormone; FT3: free triiodothyronine; FT4: free thyroxine; TT4RI: Thyrotropin T4 resistance index; TSHI: TSH index; TFQI: thyroid feedback quantile-based index

After age adjustment, in comparison to the first quartile of the TFQI (Q1), women in the third and fourth quartiles (Q3 and Q4) had a remarkably increased risk of PCOS by 130% (OR, 2.30;95% CI:1.32,4.00) and 240% (OR, 3.40;95% CI:1.87,6.18) separately (Table 2). Similarly, compared with the TSHI Q1 group, women in the Q3 and Q4 groups had a markedly increased risk of PCOS by 75% (OR, 1.75;95% CI:1.04,2.96) and 352% (OR, 4.52;95% CI:2.04,2.96) separately. Meanwhile, compared with the TT4RI Q1 group, the risk of PCOS was increased by 322% (OR, 4.22;95% CI:2.15,8.31) in the Q4 group. The FT3/FT4 quartiles have progressively increased PCOS risk (OR, 2.48 to 6.01).

**Table 2:** Associations between thyroid hormone sensitivity and PCOS

		Model 1		Model 2		Model 3	
		OR (95% CI)	*P* value	OR (95% CI)	*P* value	OR (95% CI)	*P* value
FT3/FT4 (Quartile)	Q1	1.00 (Reference)		1.00 (Reference)		1.00 (Reference)	
	Q2	2.48(1.48, 4.14)	<0.001	0.66(0.25,1.76)	0.406	0.80(0.28,2.30)	0.684
	Q3	3.09(1.80, 5.31)	<0.001	0.73(0.24,2.24)	0.580	0.71(0.21,2.45)	0.590
	Q4	6.01 (3.22, 11.21)	<0.001	0.90(0.31,2.61)	0.841	0.73(0.23,2.33)	0.590
	*P* value for trend		<0.001		0.846		0.939
TT4RI (Quartile)	Q1	1.00 (Reference)		1.00 (Reference)		1.00 (Reference)	
	Q2	0.94(0.56,1.56)	0.798	0.24(0.08,0.72)	0.010	0.27(0.09,0.82)	0.022
	Q3	1.15(0.68,1.94)	0.600	0.89(0.31,2.53)	0.822	0.92(0.30,2.79)	0.877
	Q4	4.22(2.15,8.31)	<0.001	2.88(0.86,9.70)	0.087	2.73(0.75,9.98)	0.129
	*P* value for trend		<0.001		0.002		0.007
TSHI (Quartile)	Q1	1.00 (Reference)		1.00 (Reference)		1.00 (Reference)	
	Q2	1.36(0.82,2.25)	0.238	0.96(0.35,2.62)	0.937	0.81(0.28,2.38)	0.706
	Q3	1.75(1.04,2.96)	0.036	1.57(0.57,4.30)	0.384	1.46(0.50,4.27)	0.495
	Q4	4.52(2.38,8.57)	<0.001	6.24(1.83,21.20)	0.003	5.35(1.48,19.37)	0.011
	*P* value for trend		<0.001		0.016		0.036
TFQI (Quartile)	Q1	1.00 (Reference)		1.00 (Reference)		1.00 (Reference)	
	Q2	1.17(0.71,1.93)	0.544	1.22(0.46,3.26)	0.689	1.11(0.39,3.16)	0.849
	Q3	2.30(1.32,4.00)	0.003	4.47(1.44,13.92)	0.010	3.57(1.08,11.87)	0.038
	Q4	3.40(1.87,6.18)	<0.001	5.49(1.62,18.61)	0.006	4.90(1.38,17.38)	0.014
	*P* value for trend		<0.001		0.007		0.025

Logistic regression models: Model 1 is adjusted for age; model 2 is adjusted for age and BMI; model 3 is adjusted for age, BMI, Hypertension, FPG ≥ 7.0, dyslipidemia

Hypertension was defined as SBP ≥ 140mmHg or DBP ≥ 90mmHg; Dyslipidemia was defined as TG ≥ 1.7 mmol/L or TC ≥ 5.72 mmol/L or LDL ≥ 3.1 mmol/L or HDL ≤ 0.94 mmol/L

Abbreviations: OR: odds ratio

After further adjustment for BMI, compared to the Q1 of TFQI, women in Q3 and Q4 still had a significantly increased risk of PCOS (OR 4.47,5.49, respectively); Compared with the Q1 group of TSHI, the risk of PCOS increased in Q4 (OR = 6.24); Compared with the Q1 group in TT4RI, the risk of PCOS decreased in Q2 (OR = 0.24); No difference was observed in the risk of PCOS between FT3/FT4 groups.

After further adjustment for hypertension, FPG ≥ 7.0, and dyslipidemia, compared with Q1 of TFQI, women in Q3 and Q4 remained considerably more likely to have PCOS. (OR 3.57,4.90, respectively); Q4 in TSHI showed a higher incidence of PCOS compared to Q1 (OR = 5.35); Q2 in TT4RI showed a decreased risk of PCOS compared to Q1 (OR = 0.27); No difference was observed among FT3/FT4 groups.

While finding the correlation of thyroid-related parameters with clinical, metabolic, and hormonal measures, our results revealed no significant relationship (*r* = 0.100∼0.166) (Table 3).

**Table 3:** Correlation analysis between thyroid hormone-related parameters and metabolic parameters in patients with PCOS

		TSH	FT3	FT4	FT3/FT4	TT4RI	TSHI	TFQI
HOMA-IR	r	0.080	0.092	-0.001	0.147	0.074	0.069	0.057
	*P*	0.105	0.062	0.977	0.003	0.135	0.165	0.247
T	r	-0.016	-0.031	-0.041	0.074	0.011	0.006	0.049
	*P*	0.749	0.534	0.405	0.138	0.832	0.908	0.324
FPG	r	-0.013	0.052	0.045	0.027	-0.004	0.007	0.013
	*P*	0.797	0.290	0.365	0.589	0.935	0.886	0.791
FINS	r	0.115	0.157	-0.026	0.161	0.106	0.085	0.059
	*P*	0.019	0.001	0.603	0.001	0.032	0.086	0.230
2hPG	r	-0.006	0.142	-0.024	0.149	-0.011	-0.013	-0.020
	*P*	0.910	0.004	0.634	0.002	0.830	0.786	0.685
2hINS	r	0.091	0.070	-0.030	0.091	0.081	0.066	0.043
	*P*	0.066	0.157	0.543	0.067	0.106	0.188	0.390
TG	r	0.166	0.088	-0.076	0.151	0.142	0.101	0.063
	*P*	<0.001	0.076	0.125	0.002	0.004	0.041	0.202
TC	r	0.116	-0.051	-0.090	0.026	0.093	0.060	0.023
	*P*	0.019	0.303	0.070	0.605	0.061	0.226	0.647
HDL	r	-0.132	-0.025	0.060	-0.076	-0.108	-0.067	-0.036
	*P*	0.007	0.612	0.226	0.122	0.028	0.174	0.460
LDL	r	0.100	-0.024	-0.076	0.034	0.077	0.043	0.012
	*P*	0.042	0.631	0.122	0.494	0.121	0.384	0.807

Abbreviations: HOMA-IR: homeostasis model assessment-insulin resistance; T: testosterone; FINS: fasting plasma insulin; 2hPG: 2-hour plasma glucose; 2hINS: 2-hour plasma insulin

## Discussion

In this research, we probed into the correlation among thyroid hormone sensitivity with PCOS. We found that elevated peripheral thyroid hormone sensitivity (elevated FT3/FT4) was linked to an increased PCOS risk, but there was no noticeable correlation after accounting for numerous covariates. However, diminished central thyroid hormone sensitivity (elevated TFQI, TSHI) was in connection to an elevated risk of PCOS and remained significantly associated after controlling for a few confounding factors.

Our study elucidates important associations between thyroid hormone resistance and the occurrence of PCOS. This finding provides clinicians with a deeper understanding of the underlying mechanisms involved in PCOS. Furthermore, our study sheds light on thyroid hormone sensitivity served as a potential risk indicator that can aid in early identification and early intervention of PCOS patients, thereby improving patient outcomes. More importantly, addressing thyroid hormone resistance in PCOS patients may hold therapeutic potential for improving their symptoms and overall prognosis. By improving thyroid hormone sensitivity or optimizing thyroid hormone levels, clinicians may be able to mitigate the metabolic and reproductive disturbances associated with PCOS. This could potentially lead to better outcomes, such as improved menstrual regularity, ovulation, and fertility, as well as a reduction in insulin resistance, and other metabolic abnormalities.

PCOS and thyroid diseases are both common endocrine diseases, and even though they are completely different diseases, they share quantities of clinical features, including irregular menstruation, infertility, obesity, and abnormal glucose and lipid metabolism [3, 18–20]. Although the correlation between PCOS and thyroid diseases has not been clear, several studies have confirmed that dyslipidemia and IR are more severe among sufferers with combined thyroid diseases like subclinical hypothyroidism and thyroid nodules than those with PCOS alone [21–24], implying that thyroid diseases may exacerbate metabolic disorders in PCOS patients. Nevertheless, there is still insufficient proof to say if thyroid conditions impact the onset and progression of PCOS. A population-based investigation reported that Danish PCOS females showed a considerably greater prevalence of thyroid diseases than the control group (2.5% vs. 0.7%) before the diagnosis of PCOS [25]. Two studies showed that PCOS was substantially more common in females with autoimmune thyroid illness by 39% (OR, 1.39; 95% CI:1.07,1.71) and 137% (OR, 2.37; 95% CI:1.22,4.62), separately [26, 27]. However, another study indicated that there was no difference in PCOS prevalence between euthyroid women and subclinical hypothyroidism (SCH) women, and SCH was not a standalone PCOS risk factor (OR = 0.743,95% CI 0.423–1.305) [28]. The inconsistent findings of the research may be associated with the diverse criteria used to determine thyroid status and different participating members.

Furthermore, the hypothalamus influences the pituitary gland by releasing the thyrotropin-releasing hormone (TRH), and the pituitary directs the thyroid by the release of TSH. Once there are enough thyroid hormones in the blood, they will be coupled back to the pituitary and hypothalamus through negative feedback, reducing the production and secretion of TSH and TRH. In consideration of the HPT axis’ intricate interplay, it may be insufficient for individual parameters to reflect the thyroid status. We started to inquire into the unresolved debate on the correlation between thyroid and PCOS from the aspect of thyroid hormone sensitivity adopting composed indices.

Initially, some studies proposed TSHI and TT4RI to evaluate thyroid hormone sensitivity [15, 16]. Afterwards, Laclaustra et al. [14] put forward a novel index, TFQI, which has greater stability in comparison to TSHI and TT4RI. They found that TFQI is in connection to obesity, diabetes, and MS in euthyroid people and suggested we can use this new index to detect decreased sensitivity to thyroid hormones. Several cross-sectional investigations have discovered that impaired thyroid hormone sensitivity is linked to coronary artery disease, non-alcoholic fatty liver disease, renal insufficiency, along with osteoarthritis [12, 29–31]. Similarly, our findings imply that a higher TFQI is in connection to an elevated risk of PCOS, and this correlation remains statistically meaningful when other confounding factors are taken into account. Accordingly, we hypothesize that the occurrence of PCOS may be in connection with the central sensitivity to thyroid hormones.

Previous studies have examined the potential mechanisms by which thyroid hormones affect PCOS, mainly through the following: (1) Although currently an accepted genotypic milieu has not been set up, gene polymorphisms such as FBN3 gene variants cause the pathogenesis of PCOS and HT by affecting TGFb’s activity [32, 33]. (2) Thyroid function antibodies (stimulatory or inhibitory) may interact with functional ovarian antibodies (stimulatory or inhibitory) [34]. (3) Through their impacts on FSH, LH, and GnRH levels, thyroid hormones have the power to regulate the growth of germ cells and even to disrupt the HPT axis’s ability to operate [35]. (4) In experiments involving animals, all thyroid axis receptors, such as TRH, TSH, and thyroid hormones, existed in the monkey uterus [36], and even TSH receptors, TRα1 receptors, and TRβ1 receptors were expressed in the human endometrium [37]. Of course, it cannot be excluded that the comparative deficiency of thyroid hormones due to reduced sensitivity may also influence PCOS by means of the above-mentioned mechanisms.

For all we know, this research marks the first to investigate the connection among thyroid hormone sensitivity with PCOS. Although this study is relatively novel and achieved significant results, there exist a few limitations as well. The first and foremost is data missing: gonadal hormones and insulin concentrations are not routinely measured in healthy check-up populations, so the possibility of residual confounding factors cannot be eliminated. Secondly, epidemiological surveys are subject to challenges such as genetic, environmental, as well as other variables which could affect clinical outcomes, and consequently, we should view the results with a cautious eye. Furthermore, as an inventory survey, this investigation has not been capable of identifying the fundamental causes of the phenomenon. Further prospective studies are needed to confirm the potential benefits of improving thyroid hormone resistance in PCOS patients, including symptom, reproductive function, and prognostic assessment, to provide strong evidence of its effectiveness. In addition, mechanistic studies can reveal the molecular pathways between improvement of thyroid hormone resistance and improvement of PCOS symptoms, which can enhance the understanding of PCOS pathophysiology and investigate therapeutic targets.

## Conclusion

Our research elucidates the linkage among PCOS risk in euthyroid people with diminished central sensitivity to thyroid hormones. Compared to individual parameters such as TSH and FT4, composed indices such as TFQI exhibit a stronger association with PCOS. Therefore, TFQI is expected to be a novel potential risk indicator to help clinicians early identify patients with high risk of PCOS.

Continuous variables are presented as mean ± standard deviation (SD) or median (interquartile range).

Abbreviations: BMI, body mass index; SBP, systolic blood pressure; DBP, diastolic blood pressure; FPG, fasting plasma glucose level; TG, triglycerides; TC, total cholesterol; HDL-C, high-density lipoprotein cholesterol; LDL-C, low-density lipoprotein cholesterol; TSH, thyroid stimulating hormone; FT3, free triiodothyronine; FT4, free thyroxine; TT4RI, Thyrotropin T4 resistance index; TSHI, TSH index; TFQI, thyroid feedback quantile-based index.

## Data Availability

Data and materials are available from the corresponding author on reasonable request.
